# Elevated N‐terminal prohormone of brain natriuretic peptide among persons living with HIV in a South African peri‐urban township

**DOI:** 10.1002/ehf2.12849

**Published:** 2020-06-25

**Authors:** Tess E. Peterson, Jason V. Baker, Lye‐Yeng Wong, Adam Rupert, Ntobeko A. B. Ntusi, Hanif Esmail, Robert Wilkinson, Irini Sereti, Graeme Meintjes, Mpiko Ntsekhe, Friedrich Thienemann

**Affiliations:** ^1^ Division of Epidemiology and Community Health University of Minnesota Minneapolis MN USA; ^2^ Infectious Diseases Hennepin Healthcare Research Institute Minneapolis MN USA; ^3^ Department of Medicine University of Minnesota Minneapolis MN USA; ^4^ Department of Surgery Oregon Health Sciences University Portland OR USA; ^5^ Leidos Biomedical Research Inc Frederick National Laboratory for Cancer Research Frederick MD USA; ^6^ Department of Medicine University of Cape Town Cape Town South Africa; ^7^ Wellcome Centre for Infectious Disease Research in Africa, Institute of Infectious Disease and Molecular Medicine and Department of Medicine University of Cape Town Cape Town South Africa; ^8^ MRC Clinical Trials Unit University College London London UK; ^9^ Institute for Global Health University College London London UK; ^10^ Department of Infectious Disease Imperial College London London UK; ^11^ Francis Crick Institute London UK; ^12^ Laboratory of Immunoregulation, National Institutes of Allergy and Infectious Diseases National Institutes of Health Bethesda MD USA; ^13^ Department of Medicine University Hospital Zurich Zurich Switzerland

**Keywords:** HIV infection, Cardiac stress, NT‐proBNP, South Africa

## Abstract

**Aims:**

Efforts to improve access to antiretroviral therapy (ART) have shifted morbidity and mortality among persons living with HIV (PLWH) from AIDS to non‐communicable diseases, such as cardiovascular disease (CVD). However, contemporary data on CVD among PLWH in sub‐Saharan Africa in the current ART era are lacking. The aim of this study was to assess the burden of cardiac stress among PLWH in South Africa via measurement of N‐terminal prohormone of brain natriuretic peptide (NT‐proBNP).

**Methods and results:**

NT‐proBNP was measured at baseline in 224 PLWH enrolled in a sub‐study of a tuberculosis vaccine trial in Khayelitsha township near Cape Town, South Africa. Thresholds were applied at the assay's limit of detection (≥137 pg/mL) and a level indicative of symptomatic heart failure in the acute setting (>300 pg/mL).

Mean (*SD*) age of participants was 39 (6) years, 86% were female, and 19% were hypertensive. Mean (*SD*) duration of HIV diagnosis was 8.3 (3.9) years and CD4 + count was 673 (267) with 79% prescribed ART for a duration of 5.6 (2.7) years. Thirty‐one percent of participants had NT‐proBNP > 300 pg/mL. Elevated vs. undetectable NT‐proBNP level was associated with older age (*P* = 0.04), no ART (*P* = 0.03), and higher plasma tumour necrosis factor‐α (*P* = 0.01).

**Conclusions:**

Among South African PLWH largely free of known CVD and on ART with high CD4 + counts and few comorbidities, we observed a high proportion with elevated NT‐proBNP levels, suggesting the burden of cardiac stress in this population may be high. This observation underscores the need for more in‐depth research, including the current effect of HIV on heart failure risk among a growing ART‐treated population in sub‐Saharan Africa.

## Introduction

Cardiovascular disease (CVD) is a leading cause of morbidity and mortality among persons living with HIV (PLWH) who have access to antiretroviral therapy (ART).^1^, ^2^ Extensive evidence shows that HIV infection is an independent risk factor for CVD.^3^ However, research on HIV‐associated CVD has largely been conducted in high‐income countries with an emphasis on atherosclerotic disease (e.g. risk of myocardial infarction). Nearly 70% of the global HIV epidemic exists in sub‐Saharan Africa,^4^ where access to ART is increasing^5^ and heart failure (HF) is a common CVD manifestation.^6^, ^7^, ^8^, ^9^, ^10^ Despite the high prevalence of HIV infection in countries such as South Africa (SA), the magnitude and phenotype of CVD risk among PLWH in this region remains poorly characterized in the current ART era.

N‐terminal prohormone of brain natriuretic peptide (NT‐proBNP) is a powerful clinical biomarker secreted in response to myocyte stretch due to increased ventricular preload.^11^, ^12^ It is elevated with myocardial dysfunction during both systole and diastole and is an important diagnostic test used in clinical routine to support diagnosis of both HF with reduced and preserved ejection fraction.^13^, ^14^, ^15^, ^16^ In addition to aiding in the diagnosis of HF, NT‐proBNP also improves risk prediction for CVD outcomes as well as mortality.^17^, ^18^, ^19^, ^20^, ^21^, ^22^, ^23^, ^24^, ^25^, ^26^, ^27^, ^28^, ^29^, ^30^

### Aim

The aim of this study was to assess the burden of cardiac stress via measurement of NT‐proBNP levels among a population of PLWH with few comorbidities and at low risk for AIDS.

## Methods

### Study population

A phase IIB tuberculosis (TB) vaccine trial (C‐030‐485, NCT01151189) conducted among PLWH in Khayelitsha township in SA recruited asymptomatic adults with few comorbidities and at low risk for opportunistic illness.^31^ The current study includes a subsample of participants from this vaccine trial that were enrolled in the HIV‐HEART sub‐study and gave written informed consent to storage of baseline blood specimens for future testing. The study was conducted in accordance with local and international ethics standards (UCT HREC 307/2013).

Khayelitsha is a low‐income, densely populated peri‐urban township located 35 km from Cape Town, SA with an estimated HIV prevalence of 20%.^32^ The target population for entry into the parent TB vaccine trial was PLWH age 18–50 years with CD4 + counts of >300 cells/μL if ART experienced or >350 cells/μL if ART naïve. Exclusion criteria included acute illness, fever, evidence of active TB disease, history of cancer, history of liver disease, or history of renal failure at entry—the time of blood draw for biomarker assessment. Participants were recruited from the community via radio and newspaper advertisements as well as pamphlets distributed at primary care clinics.

### Clinical data

Clinical data were collected at the time of enrolment in the vaccine trial and included both confirmed and participant self‐reported data. Confirmed data were obtained from medical records (e.g. medical diagnoses, prior clinical labs, and prescribed medications), and self‐reported data were ascertained by interview (e.g. drug use).

### Biomarker measurement

Measurement of NT‐proBNP as well as biomarkers of inflammation [interleukin‐6 and tumour necrosis factor‐α (TNF‐α)] was performed using enzyme‐linked immunosorbent assays and standardized protocols on fasting serum samples collected at parent trial baseline and stored at −70°C.

### Statistical methods

Descriptive analyses involved examination of variable distributions via histograms and frequency tables for continuous and categorical variables, respectively. NT‐proBNP levels were assessed using thresholds applied at the assay's lower limit of detection (≥137 pg/mL) and the upper limit of normal in the acute setting by European Society of Cardiology HF diagnostic guidelines (>300 pg/mL).^33^ Demographic and clinical characteristics were then compared between participants with NT‐proBNP level < 137 pg/mL and those with >300 pg/mL using complete case logistic regression. All analyses were conducted using SAS version 9.4 with a two‐sided Type I error probability of 0.05.

## Results

The C‐030‐485 trial enrolled 292 participants at the Cape Town site. Excluded from analyses were those without consent into the HIV‐HEART sub‐study or with no specimen available (*n* = 68), resulting in a final sample size of *n* = 224. Data on demographics and clinical history were missing in up to 30 participants (13%), and recent HIV viral load and CD4 + cell counts were missing in up to 75 participants (33%).

Participant demographic and clinical characteristics as well as the distribution of NT‐proBNP are presented in *Table* *1*. The overall study sample had a mean (*SD*) age of 39 (6) years and was predominantly female participants (86%). Eleven percent of participants were self‐reported current tobacco users, and 46% had history of prior TB infection. Nineteen percent of participants were diagnosed with hypertension, and the proportion with diabetes, dyslipidaemia, and known prior CVD were each 2%. One or more cardiopulmonary symptoms were observed in 10% of participants at the time of the exam, including palpitations in 2%, angina in 3%, cough in 4%, hemoptysis in 2%, dyspnoea in 2%, oedema in 2%, dizziness in 3%, syncope in 2%, and fatigue in 3%. Two or more of these symptoms were observed in 3% of participants.

**Table 1:** Demographic and clinical characteristics of study participants (*n* = 224)

	Mean (*SD*) or Proportion (*n*)^a^				High vs. low NT‐proBNP *P* value^b^
	Overall	Low NT‐proBNP < 137 pg/mL	NT‐proBNP 137 to 300 pg/mL	High NT‐proBNP > 300 pg/mL	
Participants	224	61% (136)	8% (19)	31% (69)	‐
**Demographics**					
Age, years	39 (6)	38 (6)	40 (6)	40 (6)	0.04
Female sex at birth	86% (193)	86% (117)	89% (17)	86% (59)	0.92
Black African race	100% (224)	100% (136)	100% (19)	100% (69)	‐‐
**Clinical history**					
Current tobacco user	11% (24)	10% (13)	16% (3)	12% (8)	0.69
Current alcohol user	31% (67)	58% (76)	58% (11)	68% (46)	0.17
Hypertension diagnosis	19% (36)	18% (20)	6% (1)	23% (15)	0.34
Diabetes diagnosis	2% (3)	2% (2)	0% (0)	2% (1)	0.92
Dyslipidaemia diagnosis	2% (4)	4% (4)	0% (0)	0% (0)	‐‐
Prior cardiovascular disease^c^	2% (4)	1% (1)	0% (0)	5% (3)	0.14
Prior tuberculosis	46% (88)	48% (54)	50% (8)	41% (26)	0.36
Duration of HIV diagnosis, years	8.3 (3.9)	8.3 (3.9)	8.2 (3.7)	8.4 (4.0)	0.83
Currently on ART	79% (151)	84% (95)	75% (12)	70% (44)	0.03
Duration of ART, years	5.6 (2.7)	5.7 (2.6)	6.6 (3.2)	5.1 (2.7)	0.23
**Clinical data**					
Body mass index, kg/m^2^	29.0 (7.3)	29.5 (7.7)	27.4 (6.2)	28.6 (7.0)	0.42
Systolic blood pressure, mmHg	129 (15)	129 (15)	128 (12)	130 (18)	0.92
Diastolic blood pressure, mmHg	82 (16)	81 (11)	80 (12)	84 (23)	0.21
Current cardiopulmonary symptom^d^	10% (22)	11% (15)	16% (3)	6% (4)	0.31
**Laboratory data**					
Most recent CD4 + count, cells/μL	673 (267)	689 (298)	695 (294)	641 (196)	0.30
Most recent HIV viral load undetectable	70% (105)	72% (62)	50% (6)	70% (37)	0.77
IL‐6, pg/mL	1.11 (1.99)	1.14 (1.76)	0.70 (0.39)	1.14 (2.59)	0.98
TNF‐α, pg/mL	3.20 (1.40)	2.98 (0.95)	3.37 (1.71)	3.58 (1.91)	0.008

^a^ Data on demographics and clinical history were missing in up to 30 participants, and recent HIV viral loads and CD4 + cell counts were missing in up to 75 participants. When analysis was restricted to those with complete clinical data, 60% had NT‐proBNP < 137 pg/mL, 8% were 137–300 pg/mL, and 33% were >300 pg/mL.

^b^ *P* value computed for between‐group difference in mean or proportion using univariable complete case logistic regression.

^c^ Includes *n* = 1 prior stroke; *n* = 2 prior ischaemic heart disease; and *n* = 1 prior rheumatic heart disease.

^d^ Includes palpitations, angina, cough, hemoptysis, dyspnoea, oedema, dizziness, syncope, or fatigue.

The mean (*SD*) duration of HIV diagnosis was 8.3 (3.9) years, and of those with historical HIV laboratory results available, the mean (*SD*) CD4 + count was 673 (267) cells/μL, and 70% had an undetectable HIV viral load at their most recent clinic visit. Seventy‐nine percent of participants were on ART, and of those, mean (*SD*) duration of ART treatment was 5.6 (2.7) years. Of those receiving ART, the precise regimen was known in 60%, among which the most frequently prescribed nucleoside reverse transcriptase inhibitors were emtricitabine (89% of those on ART with known regimen) and tenofovir disoproxil fumarate (64%). Non‐nucleoside reverse transcriptase inhibitors were prescribed to 93% of those on ART with known regimen, the most common of which was efavirenz, and protease inhibitors were prescribed to 8%, the most common of which was lopinavir.

Sixty‐one percent of participants had an undetectable low NT‐proBNP (i.e. <137 pg/mL), with 39% having detectable levels of NT‐proBNP ≥ 137 pg/mL. The majority of this detectable subgroup had NT‐proBNP above the clinical HF rule‐out threshold of 300 pg/mL (31% of total participants). When analysis was restricted to those with complete clinical data, results were similar: 60% had NT‐proBNP <137 pg/mL, 8% between 137 and 300 pg/mL, and 32% >300 pg/mL.

Compared with participants with NT‐proBNP < 137 pg/mL, those with values > 300 pg/mL were slightly older with a mean (*SD*) age of 38 (6) and 40 (6) years, respectively (*P* = 0.04), and had slightly higher plasma TNF‐α [3.0 (1.0) vs. 3.6 (1.9) pg/mL; *P* = 0.008]. There was no significant between‐group difference observed in the proportion of participants (86 vs. 86%; *P* = 0.92), current smokers (10 vs. 12%; *P* = 0.69), or hypertensive (18 vs. 23%; *P* = 0.34) nor in mean (*SD*) of body mass index [29.5 (7.7) vs. 28.6 (7.0) kg/m^2^; *P* = 0.42]. There were also no significant between‐group differences observed in the following HIV‐related factors: prior TB (48 vs. 41%; *P* = 0.36), undetectable HIV viral load (72 vs. 70%; P = 0.77), and mean (*SD*) CD4 + count [689 (298) vs. 641 (196) cells/μL; *P* = 0.30]. However, more participants with NT proBNP < 137 pg/mL were currently on ART compared with those with values > 300 pg/mL (84 vs. 70%; P = 0.03). The effect of no ART on elevated NT‐proBNP was independent of age and partially mediated by TNF‐α in this population.

## Conclusions

Among young and asymptomatic PLWH that presented for a vaccine trial in a peri‐urban township near Cape Town, SA, we observed a very high proportion with elevated NT‐proBNP as defined by a validated diagnostic threshold. This finding provides important and previously unavailable contemporary data that suggest a substantial potential for risk of cardiac stress and dysfunction among PLWH within sub‐Saharan Africa in the current era of widespread ART use.

Recent data from settings outside SA suggest that PLWH are at a significantly higher risk for HF with both reduced and preserved ejection fraction when compared with uninfected controls.^34^, ^35^, ^36^ HF is a heterogeneous condition that can be caused by abnormalities of the myocardium, valves, endocardium, and pericardium, all of which measurement of NT‐proBNP may detect and all of which may be of particular importance among PLWH in resource‐limited settings.^37^

There are important limitations to this study. Elevated NT‐proBNP is an indirect assessment of cardiac dysfunction, the interpretation of which depends on clinical context. A very low proportion of this population had cardiopulmonary symptoms (including dyspnoea and cough), and they largely had high CD4 + cell counts and normal levels of circulating inflammatory biomarkers. We did, however, lack other important contextual data, such as measurement of renal function. Additionally, the convenience sampling of enrolees in a separate vaccine trial may have affected generalizability of our primary observation. For example, this sample had a higher proportion of recent undetectable viral load and ART use than is estimated in the overall population of South African PLWH.^38^ Given that more advanced HIV disease with immune suppression is classically associated with myocardial dysfunction, the estimate of elevated NT‐proBNP levels we report may be conservative.

In summary, findings in this study suggest the burden of cardiac stress may be high among PWLH in SA with few underlying comorbidities. In a country where HIV prevalence is one of the highest globally and access to ART is becoming more widespread, this observation stresses the need for more in‐depth research on cardiac function in this population.

## Conflict of interest

None declared.

## FUNDING

The parent trial was supported by the European & Developing Countries Clinical Trials Partnership (IP.07.32080.002), Aeras, Bill & Melinda Gates Foundation, the Wellcome Trust (095780, 104‐803, and 203135), and the Oxford‐Emergent Tuberculosis Consortium. Laboratory work was supported by Hennepin Health Services (JVB career development award).
